# The influence of sex on activity in voluntary wheel running, forced treadmill running, and open field testing in mice

**DOI:** 10.14814/phy2.70246

**Published:** 2025-02-21

**Authors:** Adam J. Janowski, Giovanni Berardi, Kazuhiro Hayashi, Ashley N. Plumb, Joe B. Lesnak, Tahsin Khataei, Ben Martin, Christopher J. Benson, Kathleen A. Sluka

**Affiliations:** ^1^ Department of Physical Therapy & Rehabilitation Sciences University of Iowa Iowa City Iowa USA; ^2^ Department of Physical Therapy, Human Health Sciences, Graduate School of Medicine Kyoto University Kyoto Japan; ^3^ School for Behavioral and Brain Sciences and Center for Advanced Pain Studies University of Texas at Dallas Richardson Texas USA; ^4^ Department of Internal Medicine, Roy J. and Lucile A. Carver College or Medicine University of Iowa Iowa City Iowa USA; ^5^ Iowa City VA Healthcare System Iowa City Iowa USA

**Keywords:** mouse, open field, physical activity, running wheel, treadmill running

## Abstract

Physical activity is commonly used for both measuring and treating dysfunction. While preclinical work has been historically biased towards males, the inclusion of both males and females is gaining popularity. With the increasing inclusion of both sexes, it is imperative to determine sex differences in common behavioral assays. This was a secondary analysis of healthy naïve mice to determine baseline sex differences in three activity assays: voluntary wheel running (32 mice), forced treadmill running (178 mice), and open field (88 mice). In voluntary wheel running, females showed greater distance run, running time, bout duration, and speed, but no difference in total bouts. In forced treadmill running, females showed greater time to exhaustion, but no difference in maximum speed attained. In open field, males showed greater active time but no difference in distance and speed over 30 min; however, male mice showed a downward trajectory in distance and speed over the final 20 min of testing, whereas females did not. These data suggest that male mice demonstrate comparable activity intensity to female mice but do not match females' duration of activity, especially for volitional tasks. Researchers utilizing these assays should account for sex differences as they could mask true findings in an experiment.

## 1 INTRODUCTION

The World Health Organization defines physical activity as any type of bodily movement produced by skeletal muscles that requires the expenditure of energy (WHO, 2024). This broad‐reaching definition includes activities as small as short periods of standing or walking, all the way to the intense exercise performed by elite athletes. Physical activity is effective in the primary and secondary prevention of chronic diseases (Anderson & Durstine, 2019; Bushman, 2020; Warburton, 2006) and elicits changes in multiple biological systems (Lesnak et al., 2022; Lesnak, Berardi, & Sluka, 2023; Lesnak, Hayashi, et al., 2023; Lesnak & Sluka, 2020; Voss et al., 2020). Physical activity levels can also be used as a test of functional ability, allowing researchers to determine differences in physical ability between populations, as well as changes in activity due to specific interventions (Chaudhry et al., 2020; Jones et al., 2013; Matos Casano & Anjum, 2023).

Sex‐specific differences in physical activity and performance‐based tests in human subjects are well known, with females generally demonstrating reduced fatigability compared to male counterparts (Ansdell et al., 2020; Hunter, 2016; Lanning et al., 2017; McCarthy & Warne, 2022; Senefeld et al., 2013). Historically, there has existed a strong sex bias in preclinical research, evidenced by more frequent utilization of male animals relative to females. As the inclusion of both sexes in preclinical research becomes more common, it is important to determine inherent sex differences in common preclinical behavioral assays. Greater resolution of the sex‐specific differences in rodents will allow parallel studies and better translation between humans and animals.

Three commonly utilized activity assays in rodents are voluntary wheel running, forced treadmill running, and open field testing, and are used to measure the impact of pain, fatigue, motivation, and psychological states (Dougherty et al., 2016; Kraeuter et al., 2019; Manzanares et al., 2018; Wolff et al., 2017; Wolff et al., 2018). Voluntary wheel running assesses self‐selected activity in a stress‐free home environment removed from tester influence (Goh & Ladiges, 2015); forced treadmill running to exhaustion assesses maximal exercise performance (Dougherty et al., 2016; Khataei et al., 2021); and open field testing assesses self‐selected exploratory behavior in a novel environment (Prut & Belzung, 2003). The results obtained from each are uniquely meaningful. Prior work shows female animals run a greater distance and spend more time on a running wheel than males. However, the data regarding sex‐specific differences in speed, bouts, and duration in running wheels exhibit mixed results, while forced treadmill running and open field testing show multiple outcomes for various measures (Basso & Morrell, 2017; Bowen et al., 2016; De Bono et al., 2006; Dougherty et al., 2016; Fritz et al., 2017; Holcomb et al., 2022; Konhilas et al., 2004; Liu et al., 2022; Milner & Crabbe, 2008; O'Leary et al., 2013; Oydanich et al., 2019; Sun et al., 2018; Zeng et al., 2023). Thus, a better understanding of sex‐specific differences in activity measures are needed.

Advances in automated data collection and analysis allow examination of multiple indices of physical activity that may provide greater insight into different aspects of activity and function, thereby allowing for greater translation from preclinical to clinical work (De Bono et al., 2006; Goh & Ladiges, 2015). For example, individuals with chronic pain show decreased total activity and lower peak activity with the use of activity monitors (voluntary physical activity), but demonstrate similar functional capacity with controlled performance‐based tests (Kop et al., 2005; Merriwether et al., 2018). Activity measures in animals are designed to assess motivational, cognitive, and emotional states (Häger et al., 2018; Kandasamy et al., 2016; Pagliusi et al., 2020) making detailed delineation of sex differences among these assays important for study design, analysis, and interpretation. Therefore, the purpose of this study was to examine sex differences in three common activity‐based measures: voluntary wheel running, forced treadmill running, and open field exploratory behavior.

## 2 MATERIALS AND METHODS

**FIGURE 1:**
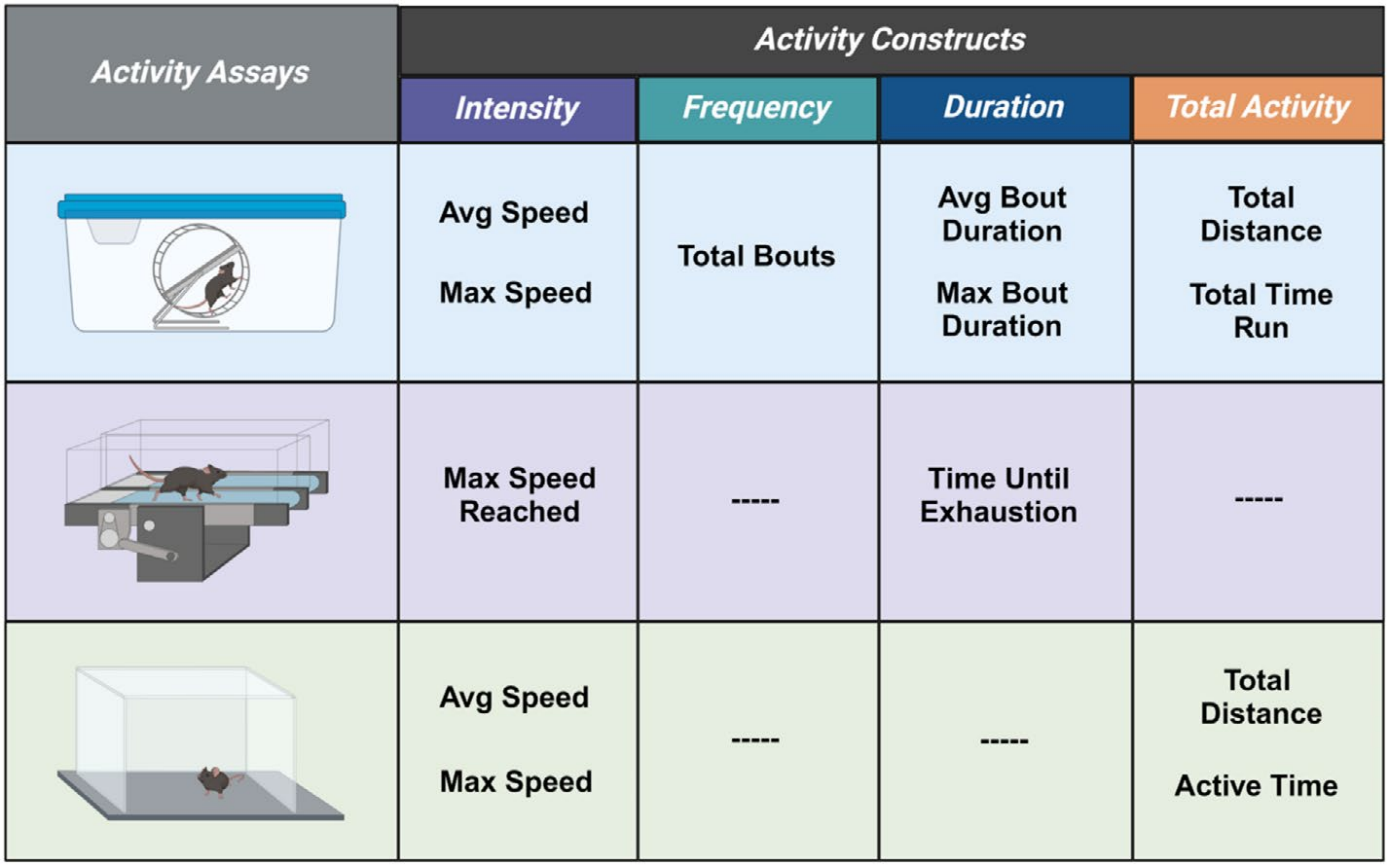
Schematic diagram showing three different activity assays. Three activity assays were utilized to investigate sex differences: Voluntary wheel running, forced treadmill running, and open field testing. Assays were broken down into different activity constructs to analyze differences with greater granularity. Graphic designed in Biorender.

This study was a secondary analysis of data that was accumulated throughout multiple experiments. Each assay was the first test performed on the individual animals as part of different experimental protocols and was performed prior to any drug or behavioral intervention. Three assays were utilized, including voluntary wheel running, forced treadmill running, and open field (Figure 1). Baseline performance measures were utilized in the case of treadmill running and open field testing, while 4 weeks of free wheel access were utilized for voluntary wheel running. Male (*n* = 122) and female (*n* = 166) mice utilized in all experiments were C57BL/6J aged 8–11 weeks (Jackson Laboratories, Bar Harbor, ME, USA). Separate animals were used for each assay. All mice were housed on a 12‐h light–dark cycle with access to food (Inotiv Teklad 7913) and water ad libitum. All experiments were approved by the University of Iowa Animal Care and Use Committee and were conducted in accordance with the National Institute of Health's Guidelines for the Care and Use of Laboratory Animals.

### 2.1 Voluntary wheel running

Voluntary wheel running was tested using 32 mice (16 male and 16 female). Mice were run in four separate cohorts of eight (4M/4F) at a time. Mice were individually housed on the first day of testing, and running wheels were immediately placed in each home cage (Columbus Instruments 0294‐4019). No formal acclimation period was provided. Running wheel activity was recorded for 28 days. On day 14, home cages and running wheels were changed. Revolutions were compiled to a spreadsheet every minute using Columbus Instrument Windows software. Because mice run primarily during the night cycle, only the 12‐h night cycle running period was used in analysis (Goh & Ladiges, 2015; Manzanares et al., 2018). Data output was analyzed with a custom python script which calculated total distance run per 12‐h night cycle (distance), percent of total minutes where running occurred (total time run), average wheel revolutions during minutes where running occurred (average speed), and the maximum revolutions in 1 min (max speed). The total number of running bouts, average duration of each running bout, and the duration of the longest running bout were also calculated. A bout of running was defined as a period of consecutive minutes where any amount of running occurred. If running stopped long enough for 1 recorded minute to pass, then a bout was considered terminated. The average bout duration was determined as the average length of each bout, and the max bout duration was the longest recorded bout. All measures of wheel revolutions were converted to distance in meters for further analysis (running wheel circumference = 11.8 inches).

### 2.2 Treadmill

Maximal treadmill running duration was tested utilizing 168 mice (62 male and 106 female). The treadmill protocol has been described previously (Khataei et al., 2021). Briefly, mice underwent a 5‐day acclimation period on the treadmill (Columbus Instruments) for 30 min daily with gradual increases in speed and incline. Electrical shock grids (1 mA, 1 Hz, 200 ms duration) were present on the rear of the treadmill to motivate maximal ambulatory time and speed during acclimation days. On testing day, the treadmill was set to a 20‐degree incline and shock grids were turned off. Mice were given a 10‐min period to acclimate to the treadmill after which they were given a 10‐min warmup at 6 m/min. Speed was then increased to 8 m/min for 3 min and subsequently increased by 2 m/min every 3 min until the mouse reached exhaustion. Shock bars remained off during testing, but gentle prodding was used with a tongue depressor to encourage running. Exhaustion was determined as the point where mice resisted prodding for 10 consecutive seconds. Total running time was recorded and analyzed. Additionally, maximum speed attained by the animal was analyzed using a subset of animals (32M, 82F) for whom that data was documented.

### 2.3 Open field

Open field testing was performed in 88 mice (44 male and 44 female). Mice were placed in one of four 16‐inch × 16‐inch open‐topped boxes with translucent walls. Solid barriers were present between cages so mice could not see each other. Recordings were completed without an investigator in the room. All testing was performed between 11 am and 2 pm to minimize the impact of time of day on data collection. Lumen level was consistent at ~140 lux during testing and throughout the middle and corners of all individual boxes. Box floors were cleaned thoroughly between each bout of testing.

Activity in the open field was recorded over 30 min at 1‐s intervals using an overhead camera (Panasonic WV‐BP334) and Limelight tracking and analysis software (Version 2.7). The 30‐min testing period was evaluated in total as well as in 5‐min intervals using a custom python script to determine distance run, active time, average speed, and max speed. Max speed was determined as the average of the top 10 fastest seconds run. Whole cage activity was used for activity measures and not separated into peripheral and central activity zones. We also performed a separate analysis of percent time in center, as a measure of anxiety, for comparison (Prut & Belzung, 2003).

### 2.4 Statistical analysis

Voluntary running wheel data was analyzed using linear mixed effects models. The primary outcomes (i.e., distance, time run, etc.) were modeled as a function of sex, time, and sex*time interaction. Two unique models were constructed for each measurement; the first model included days 1–14 as time points, and the second model included days 15–28. Previous research and unpublished data from our laboratory suggest that mice require roughly 2 weeks to reach a normalized daily running distance, but with noted variability (Basso & Morrell, 2017; Bowen et al., 2016; De Bono et al., 2006; Konhilas et al., 2004). Therefore, the first model (days 1–14) was arranged specifically to measure sex differences in day 1 running as well as the rate of change (slope) in running over the first 14 days. The second model (days 15–28) was analyzed similarly, examining sex differences in wheel running on day 15 and the rate of change from days 15 to 28; days 15 to 28 were after habituation had occurred. Each model was tested for the inclusion of random slopes and random intercepts. If the addition of random slopes proved statistically significant via partial *F*‐test, then random slopes and intercepts were utilized. Otherwise, only random intercepts were utilized. Model fit was validated using QQ plots and residual plots.

Voluntary wheel running was also analyzed by calculating the area under the curve (AUC) over the full 28‐day period. Voluntary wheel running AUC along with forced treadmill running, and open field data were first tested for normal distribution using the Shapiro‐Wilks test. *T*‐tests and Cohen's D effect sizes were used to analyze sex differences in voluntary wheel running AUC, forced treadmill running (time to exhaustion and max speed), and open field testing (total distance run, active time, average speed, and max speed over 30 min). Open field data was segmented into 5‐min intervals for time‐based longitudinal analysis. These changes in open field distance, active time, average speed, and max speed over time were analyzed using linear mixed effects models. The analysis involved splitting the measurements into the first 10 min and final 20 min, using separate models to determine initial running differences in main effects and interactions, as well as trends throughout the assay akin to the voluntary wheel running analysis above. To account for multiple comparisons, statistical tests for each assay were adjusted using the Benjamini–Hochberg method to control the false discovery rate (Benjamini & Hochberg, 1995). This adjusted value is presented as a *q*‐value. Both *q*‐values and *p*‐values are presented in Tables 1, 2, 3. Only *q*‐values are presented in the text of the article, and a *q* < 0.05 is considered significant.

**TABLE 1:** Results from the voluntary wheel running linear mixed effects models.

Variable	Days 1–14						Days 15–28					
	Effect	*β*	SE	*t*	*p*	*q*	Effect	*β*	SE	*t*	*p*	*q*
Distance (m)	Intercept	1698.94	398.58	4.26	<0.001	–	Intercept	5220.76	1253.50	4.17	<0.001	–
	Sex	1459.31	563.13	2.59	0.015	**0.034**	Sex	5842.21	1774.58	3.29	0.003	**0.009**
	Time	281.27	54.00	5.21	<0.001	**<0.001**	Time	13.31	40.04	0.33	0.742	0.826
	Sex*Time	320.33	76.20	4.20	<0.001	**0.002**	Sex*Time	−70.77	56.73	−1.25	0.222	0.351
Total time run (%)	Intercept	24.46	3.11	7.88	<0.001	–	Intercept	33.88	2.96	11.43	<0.001	–
	Sex	14.35	4.39	3.27	0.003	**0.009**	Sex	22.17	4.20	5.28	<0.001	**<0.001**
	Time	0.94	0.25	3.75	0.001	**0.004**	Time	0.00	0.11	−0.01	0.992	0.992
	Sex*Time	0.19	0.35	0.54	0.596	0.733	Sex*Time	−0.36	0.15	−2.44	0.015	**0.034**
Average speed (m/min)	Intercept	7.70	0.98	7.85	<0.001	–	Intercept	16.72	2.05	8.18	<0.001	–
	Sex	0.12	1.39	0.08	0.935	0.954	Sex	3.70	2.89	1.28	0.211	0.345
	Time	0.68	0.09	7.19	<0.001	**<0.001**	Time	0.04	0.07	0.59	0.557	0.718
	Sex*Time	0.49	0.13	3.68	0.001	**0.004**	Sex*Time	0.03	0.09	0.28	0.780	0.831
Max speed (m/min)	Intercept	17.34	1.84	9.42	<0.001	–	Intercept	34.16	1.74	19.63	<0.001	–
	Sex	−0.58	2.60	−0.22	0.826	0.861	Sex	1.48	2.46	0.60	0.553	0.718
	Time	1.18	0.14	8.29	<0.001	**<0.001**	Time	0.08	0.06	1.39	0.176	0.309
	Sex*Time	0.58	0.20	2.88	0.007	**0.019**	Sex*Time	0.11	0.09	1.29	0.206	0.345
Total bouts	Intercept	53.76	4.34	12.38	<0.001	–	Intercept	58.13	3.17	18.35	<0.001	–
	Sex	2.85	6.14	0.46	0.646	0.772	Sex	−1.37	4.49	−0.31	0.761	0.829
	Time	0.29	0.41	0.71	0.485	0.660	Time	−0.20	0.11	−1.90	0.059	0.111
	Sex*Time	−0.24	0.58	−0.41	0.684	0.779	Sex*Time	0.15	0.15	0.96	0.339	0.512
Average bout time (min)	Intercept	4.13	0.47	8.88	<0.001	–	Intercept	5.34	0.88	6.06	<0.001	–
	Sex	1.41	0.66	2.14	0.041	0.083	Sex	4.21	1.25	3.38	0.002	**0.008**
	Time	0.14	0.05	2.86	0.008	**0.019**	Time	0.02	0.03	0.71	0.485	0.660
	Sex*Time	0.13	0.07	1.89	0.068	0.124	Sex*Time	−0.09	0.04	−1.99	0.056	0.110
Max bout time (min)	Intercept	13.61	2.43	5.61	<0.001	–	Intercept	20.77	3.86	5.39	<0.001	–
	Sex	10.32	3.43	3.01	0.005	**0.015**	Sex	12.11	5.47	2.21	0.028	0.059
	Time	0.79	0.26	3.00	0.005	**0.015**	Time	0.14	0.15	0.95	0.345	0.512
	Sex*Time	0.15	0.37	0.42	0.680	0.779	Sex*Time	−0.11	0.22	−0.53	0.598	0.733

*Note*: Each running wheel construct had two regressions performed: the first from day 1 to 14 and the second from day 15 to 28. Each model included a term for sex, time, and sex*time interaction. *q* < 0.05 statistically significant (bold).

Abbreviations: m, meters; min, minute; q, Benjamini–Hochberg multiple comparisons correction; SE, standard error.

## 3 RESULTS

### 3.1 Voluntary wheel running task

#### 3.1.1 Total activity volume: Total distance and total time run

##### Distance

**FIGURE 2:**
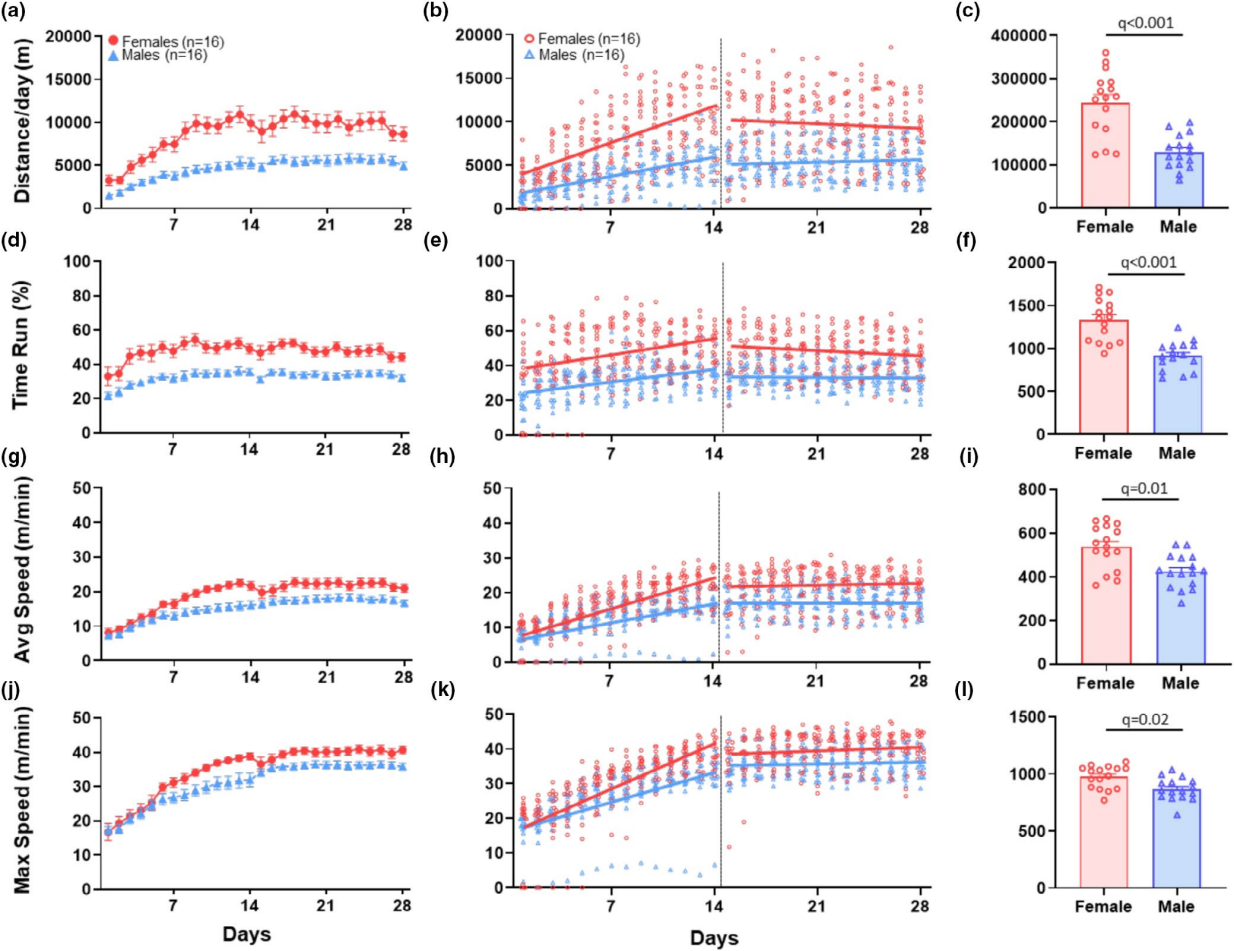
Running wheel behavior—distance, time, and speed. Results from voluntary wheel running analysis of distance (a–c), total time run (d–f), average speed (g–i) and max speed (j–l). Graphs in the left column (a, d, g, j) show daily data over the 28‐day time period for male and female mice. Data are represented as the mean + SEM. Graphs in the middle column (b, e, h, k) show individual data points for each animal and the regression lines for the acclimation phase, Days 1–14, and for the plateau phase, Days 15–28. The right column (c, f, i, l) shows summary bar graphs for the AUC for the entire 28‐day period. Data are represented as the mean + SEM. The adjusted *p* value (*q*) considered significant if <0.05.

On the first day in running wheels, females ran an average distance of 3158 m compared to males who ran 1699 m (sex effect: *β* = 1459, *q* = 0.034) (Figure 2a,b, Table 1). Over the first 14 days, both males and females continued to increase their running distance, with females increasing their distance at a greater rate; females increased at 501 m/day and males 281 m/day (time effect: *β* = 281, *q* < 0.001; sex*time: *β* = 320, *q* < 0.002) (Figure 2a,b, Table 1). During days 15–28, females ran an average distance of 11,062 m/day compared to males at 5221 m/day (sex effect: *β* = 5842, *q* < 0.009) (Figure 2a,b, Table 1). AUC over the entire 28‐day period showed that females (243 km ± 77) ran a greater distance than males (134 km ± 37) (*q* < 0.001, *δ* = 1.8) (Figure 2C, Table 2).

**TABLE 2:** Results from voluntary wheel running, forced treadmill running, and open field.

Test	Females (mean ± SD)	Males (mean ± SD)	*p*	*q*	*d*
Running wheels					
Distance (m)	243414.5 ± 77327.0	134302.4 ± 36544.8	<0.001	**<0.001**	1.8
Percent minute run (AUC %)	1328.5 ± 253.9	928.9 ± 156.7	<0.001	**<0.001**	1.89
Average meters/minute (m)	538.5 ± 104.9	432.8 ± 70.0	0.003	**0.011**	1.19
Max meters/minute (m)	982.2 ± 98.8	872 ± 98.9	0.005	**0.015**	1.11
Running bouts	1598.9 ± 235.3	1529.6 ± 198.8	0.391	0.563	0.32
Average bout time (min)	215.9 ± 54.5	156.7 ± 29.1	0.001	**0.006**	1.36
Max bout time (min)	883.9 ± 223.2	618.7 ± 143.8	<0.001	**0.004**	1.41
Treadmill					
Running duration (min)	36.79 ± 3.59	35.29 ± 2.58	0.002	**0.004**	0.46
Max velocity reached (m/min)	22.4 ± 2.27	21.6 ± 1.81	0.052	0.052	0.37
Open field					
Distance (m)	103.95 ± 14.51	101.43 ± 12.13	0.379	0.523	0.19
Active time (s)	1608.55 ± 47.03	1646.75 ± 43.56	<0.001	**<0.001**	0.84
Average velocity (m/min)	3.88 ± 0.51	3.69 ± 0.42	0.073	0.133	0.39
Max velocity (m/min)	14.29 ± 1.21	13.80 ± 1.06	0.045	0.087	0.43
Percent in center (%)	15.2 ± 4.8	14.5 ± 4.3	0.664	0.741	0.14

*Note*: Results include AUC values of the seven different voluntary wheel running constructs, as well as forced treadmill running duration and max velocity, and results from the whole 30‐min open field test. Sex differences were assessed using *t*‐tests comparing males and females, adjustment for multiple comparisons (*q*), and effect sizes (Cohen's D). Data presented as means ± SD. *q* < 0.05 statistically significant (bold).

Abbreviations: AUC, area under curve; d, Cohen's D; m, meters; min, minute; s, seconds; SD, standard deviation.

##### Time

On day 1, females ran an average of 39% of the time, whereas males ran an average of 25% of the time (sex effect: *β* = 14.35, *q* = 0.009) (Figure 2d,e, Table 1). Both males and females increased total run time by ~1% per day over 14 days with no difference in the rate of increase. Days 15–28, females ran on average 56% of the time and males 33% of the time (sex effect: *β* = 22.17, *q* < 0.001) (Figure 2d,e, Table 1). AUC over the entire 28‐day period showed that females (1329 ± 253) ran a greater percent of the time than males (929 ± 157) (*q* < 0.001, *δ* = 1.80) (Figure 2F, Table 2).

#### 3.1.2 Activity intensity: Average and peak speed

On day 1 in running wheels, there was no difference between males and females in average speed (sex effect: *β* = 0.12, *q* = 0.95) (Figure 2g,h, Table 1) or max speed (sex effect: *β* = −0.58, *q* = 0.86) (Figure 2j,k, Table 1). During the first 14 days, males increased average speed daily by 0.68 m/min (time effect: *β* = 0.68, *q* < 0.001) (Figure 2g,h, Table 1) and females by 1.17 m/min each day (sex*time: *β* = 0.49, *q* = 0.004) (Figure 2g,h, Table 1). Males increased max speed an average of 1.18 m/min each day (time effect: *β* = 1.18, *q* < 0.001) (Figure 2j,k, Table 1) and females 1.76 m/min (sex*time: *β* = 0.58, *q* = 0.019) (Figure 2j,k, Table 1). During days 15–28, males and females achieved daily average speeds of 16.7 and 20.4 m/min, respectively (sex effect: *β* = 3.70, *q* = 0.35) (Figure 2g,h, Table 1) and max speeds of 34.2 and 35.7 m/min (sex effect: *β* = 1.48, *q* = 0.71) (Figure 2j,k, Table 1). These differences were not statistically significant. However, AUC over the entire 28‐day period showed that females ran greater average (539 ± 105) and max (982 ± 99) speed than males (432 ± 70) (872 ± 99) (average: *q* < 0.011, *δ* = 1.19) (max: *q* < 0.015, *δ* = 1.11) (Figure 2i,j, Table 2).

#### 3.1.3 Frequency and duration of activity: Bout number and duration

**FIGURE 3:**
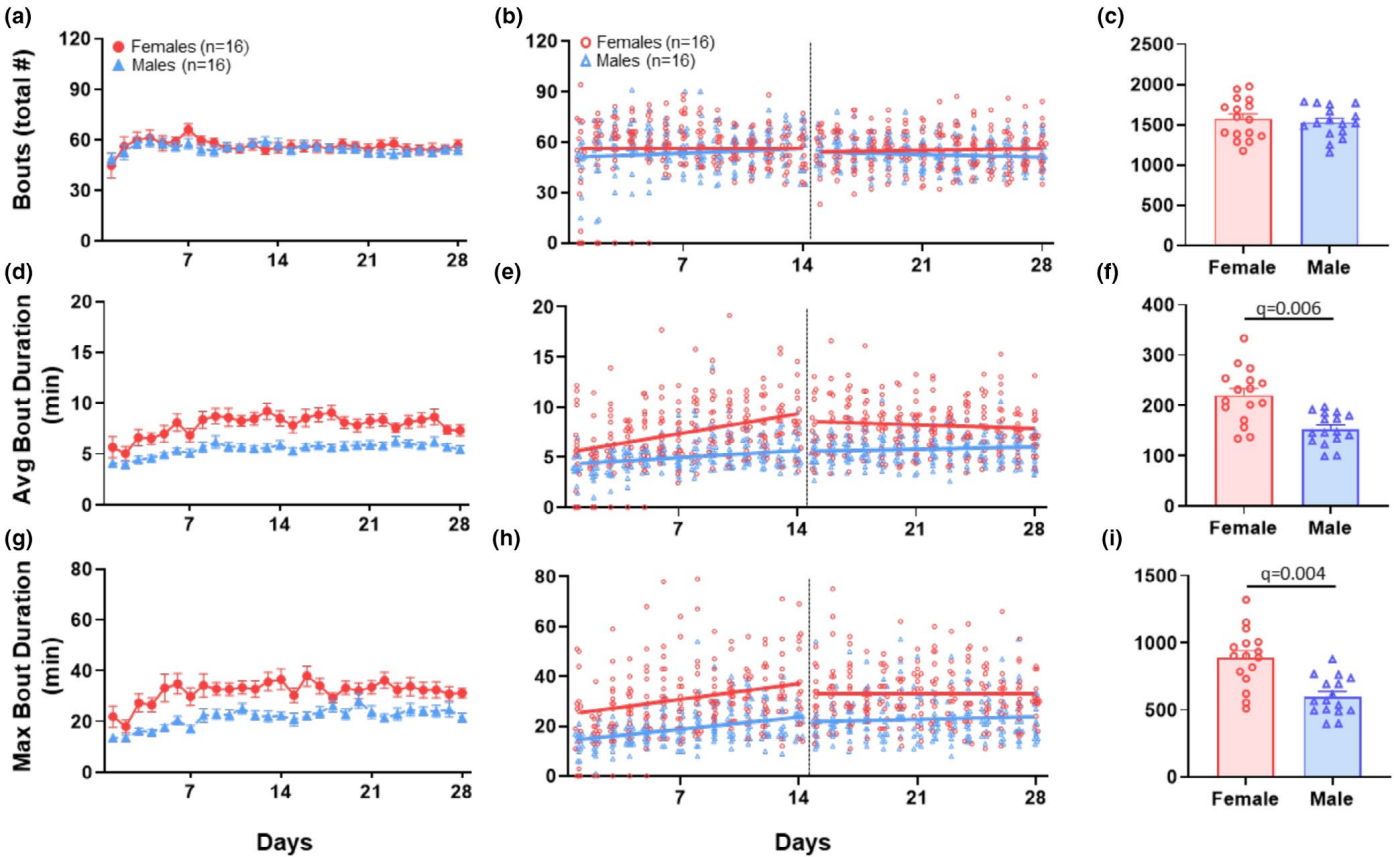
Running wheel behavior—bouts. Results from voluntary wheel running analysis of total bouts (a–c), average bout time (d–f), and max bout time (g–i). Graphs in the left column (a, d, g) show daily data over the 28‐day time period for male and female mice. Data are represented as the mean + SEM. Graphs in the middle column (b, e, h) show individual data points for each animal and the regression lines for the acclimation phase, Days 1–14, and for the plateau phase, Days 15–28. The right column (c, f, i) shows summary bar graphs for the AUC for the entire 28‐day period. Data are represented as the mean + SEM. The adjusted *p* value (*q*) considered significant if <0.05.

On day 1 in running wheels, there was no sex difference in total bouts run (sex effect: *β* = 2.85, *q* = 0.77) (Figure 3a,b, Table 1) or average bout duration (sex effect: *β* = 1.41, *q* = 0.083) (Figure 3d,e, Table 1); however, females had a longer max bout duration (sex effect: *β* = 10.32, *q* = 0.015) (Figure 3g,h, Table 1) with an average of 23.9 min compared to males, who averaged 13.6 min. During days 1–14, there was no difference in the rate of increase of bouts run (sex*time: *β* = −0.24, *q* = 0.78) (Figure 3a,b, Table 1), average bout duration (sex*time: *β* = 0.13, *q* = 0.12) (Figure 3d,e, Table 1), or max bout duration (sex*time: *β* = 0.15, *q* = 0.78) (Figure 3g,h, Table 1) between sexes. During days 15–28, females ran 56.8 total bouts and males 58.1 total bouts on average, which was not a statistically significant difference (sex effect: *β* = −1.37, *q* = 0.83) (Figure 3a,b, Table 1). Also, during days 15–28, female's average daily bout duration was 9.6 min compared to 5.3 min for males (sex effect: *β* = 4.21, *q* = 0.008) (Figure 3d,e, Table 1) and female's daily max bout duration was 32.9 min compared to 20.8 min for males (sex effect: *β* = 12.11, *q* = 0.059) (Figure 3g,h, Table 1). AUC over the entire 28‐day period showed that females (1599 ± 235) ran the same number of bouts as males (1530 ± 199) (*q* = 0.56, *δ* = 0.32) (Figure 3c, Table 2), while it showed that females had a greater average (216 ± 55) and max (884 ± 223) bout duration than males (157 ± 29) (619 ± 144) (average: *q* = 0.006, *δ* = 1.36) (max: *q* = 0.004, *δ* = 1.41) (Figure 3f,i, Table 2).

#### 3.1.4 Forced treadmill running (maximum exercise capacity)

**FIGURE 4:**
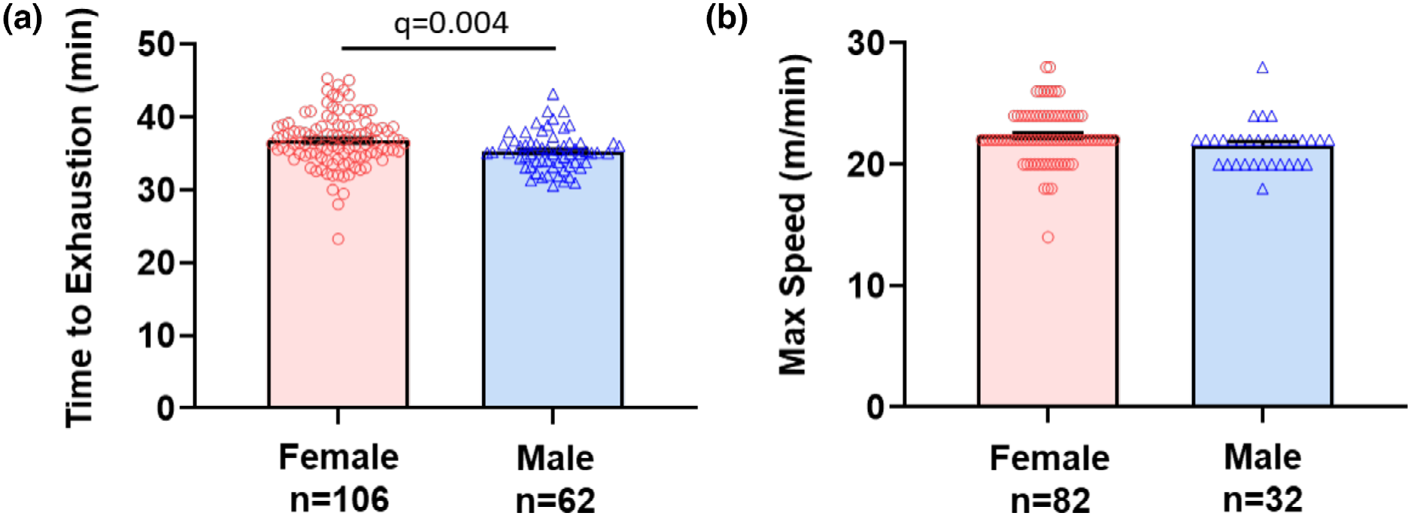
Forced treadmill running. Bar graphs and individual data points for male and female mice for (a) time to exhaustion and (b) maximum speed of running reached. Data are represented as the mean ± SEM. The adjusted *p* value (*q*) considered significant if <0.05.

During the forced treadmill task, males ran an average of 35.3 min and females ran 36.8 min, which was significantly different (*p* = 0.004, *δ* = 0.46) (Figure 4a, Table 2). The maximum speed attained during the exercise protocol was no different between males (21.60 ± 1.81 m/min) and females (22.40 ± 2.27 m/min) (*p* = 0.052, *δ* = 0.37) (Figure 4b, Table 2). Thus, females had a longer time till exhaustion compared with males, but the sexes did not differ in maximum speed attained.

#### 3.1.5 Open field testing: Spontaneous exploratory behavior in a novel environment

##### Distance

**FIGURE 5:**
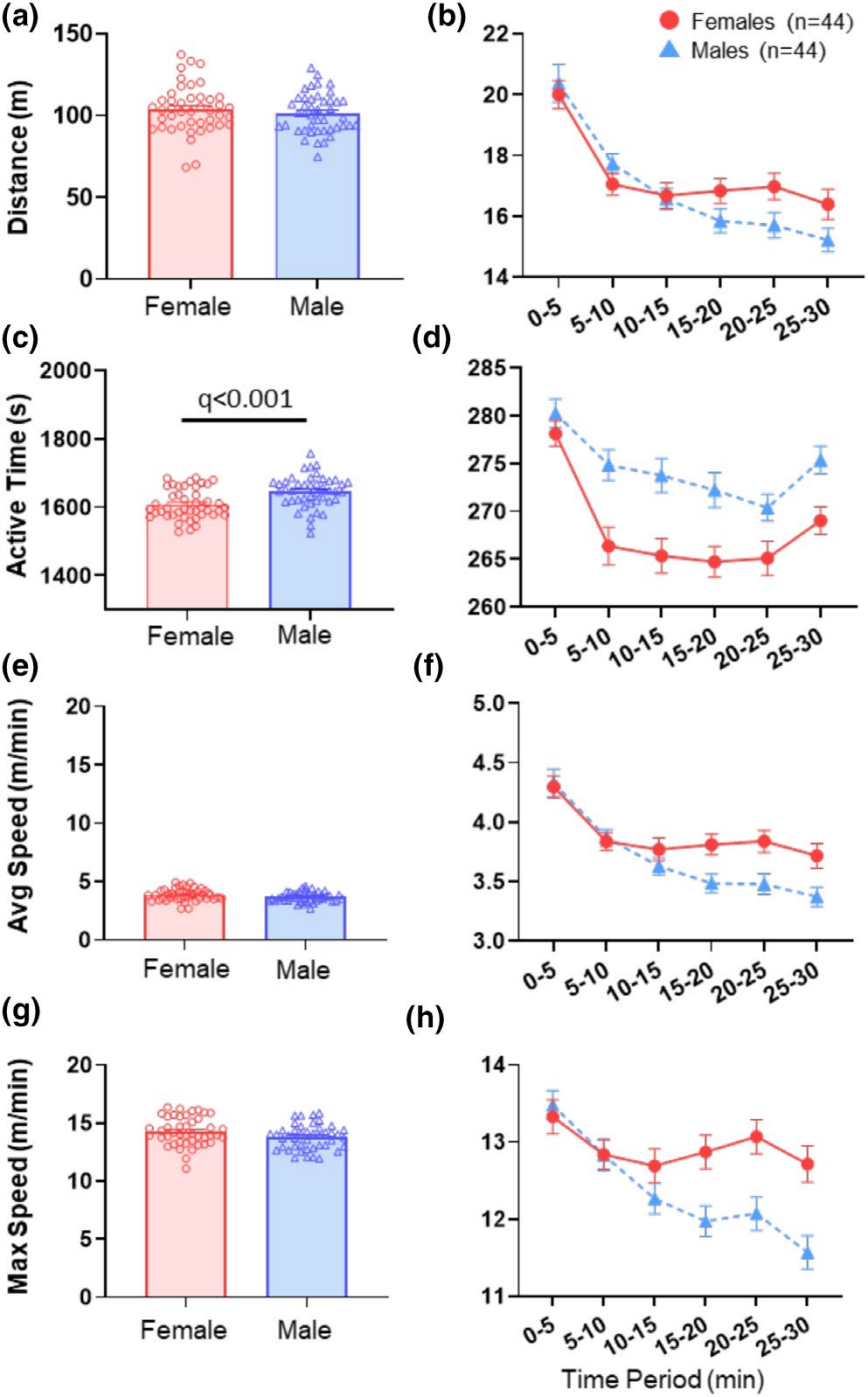
Open field test. (a) Bar graphs and individual data points for total distance traveled during the 30‐min period for female and male mice. (b) Line graph showing distance run over the 30‐min recording session broken into 5‐min intervals. (c) Bar graphs and individual data points for time spent moving during the 30‐min period for female and male mice. (d) Line graph showing time spent moving over the 30‐min recording session broken into 5‐min intervals of time. (e) Bar graphs and individual data points for average speed over the 30‐min period while moving for female and male mice. (f) Line graph showing average speed over the 30‐min recording session broken into 5‐min intervals of time. (g) Bar graphs and individual data points for maximum speed achieved during the 30‐min period for female and male mice. (h) Line graph showing maximum speed achieved in each of 5‐min intervals over the 30‐min period. Data are the mean + SEM. The adjusted *p* value (*q*) considered significant if <0.05.

Over the course of 30 min in the open field, males ran a similar average distance (101.43 m ± 12.13) compared to females (103.95 m ± 14.51) (*q* = 0.52, *δ* = 0.19) (Figure 5a, Table 2). When examining 5‐min segments, both sexes equally decreased distance in the first 10 min (time effect: *β* = −2.64, *q* < 0.001) (sex*time: *β* = −0.31, *q* = 0.74) (Figure 5B, Table 3). However, in the final 20 min, the distance run by male mice steadily decreased in distance (time effect: *β* = −0.59, *q* < 0.001) relative to females (sex*time: *β* = 0.49, *q* = 0.002) (Figure 5B, Table 3).

**TABLE 3:** Results from the open field test linear mixed‐effects models.

	0–10 min						10–30 min					
Variable	Effect	*β*	SE	*t*	*p*	*q*	Effect	*β*	SE	*t*	*p*	*q*
Distance (m)	Intercept	22.99	0.80	28.73	<0.001	–	Intercept	18.56	0.45	41.62	<0.001	–
	Sex	−0.06	1.13	−0.05	0.961	0.995	Sex	−1.37	0.63	−2.18	0.032	0.066
	Time	−2.64	0.46	−5.68	<0.001	**<0.001**	Time	−0.59	0.10	−6.05	<0.001	**<0.001**
	Sex*Time	−0.31	0.66	−0.47	0.639	0.741	Sex*Time	0.49	0.14	3.54	<0.001	**0.002**
Active Time (s)	Intercept	285.6	3.03	94.22	<0.001	–	Intercept	274.24	2.35	116.49	<0.001	–
	Sex	4.25	4.29	0.99	0.324	0.469	Sex	−10.16	3.33	−3.05	0.003	**0.008**
	Time	−5.41	1.81	−2.98	0.004	**0.009**	Time	−0.23	0.42	−0.55	0.583	0.726
	Sex*Time	−6.36	2.57	−2.48	0.015	**0.034**	Sex*Time	0.74	0.60	1.24	0.217	0.350
Average Speed (m/min)	Intercept	4.79	0.16	30.31	<0.001	–	Intercept	4.02	0.09	42.82	<0.001	–
	Sex	−0.03	0.22	−0.14	0.890	0.956	Sex	−0.16	0.13	−1.21	0.230	0.351
	Time	−0.46	0.09	−5.01	<0.001	**<0.001**	Time	−0.11	0.02	−5.58	<0.001	**<0.001**
	Sex*Time	0.00	0.13	−0.01	0.995	0.995	Sex*Time	0.10	0.03	3.35	0.001	**0.004**
Max Speed (m/min)	Intercept	14.13	0.36	39.16	<0.001	‐‐‐	Intercept	13.23	0.25	52.16	<0.001	–
	Sex	−0.31	0.51	−0.61	0.544	0.717	Sex	−0.45	0.36	−1.25	0.216	0.350
	Time	−0.65	0.21	−3.05	0.003	**0.008**	Time	−0.27	0.05	−5.17	<0.001	**<0.001**
	Sex*Time	0.16	0.30	0.53	0.601	0.726	Sex*Time	0.29	0.07	3.83	<0.001	**0.001**

*Note*: Open field data, measured over 30‐min was broken down into 5‐min increments. Each activity measure then had two regressions performed: the first analyzing sex difference from 0 to 10 min and the second from 10 to 30 min. Each model included a term for sex, time, and sex*time interaction. *q* < 0.05 statistically significant (bold).

Abbreviations: m, meters; min, minute; q, Benjamini–Hochberg multiple comparisons correction; SE, standard error.

##### Active time

Over the course of 30 min, males demonstrated more active time in the open field (1646.75 s ± 43.56) on average compared to females (1608.55 s ± 47.03) (*q* < 0.001, *δ* = 0.84) (Figure 5c, Table 2). When breaking down active time into 5‐min segments, both sexes decreased active time over the first 10 min (time effect: *β* = −5.41, *q* = 0.009) (Figure 5d, Table 3) with females decreasing more than males (sex*time: *β* = −6.36, *q* = 0.034) (Figure 5d, Table 3). Over the final 20 min in the open field, males had more active time than females (sex effect: *β* = −10.16, *q* = 0.008) (Figure 5d, Table 3) but with no significant time effect or sex*time interaction (time effect: *β* = −0.23, *q* = 0.73) (sex*time effect: *β* = 0.74, *q* = 0.35) (Figure 5D, Table 3).

##### Average speed

Males and females demonstrated no difference in average speed throughout 30 min, with males running on average 3.69 m/min and females 3.88 m/min (*q* = 0.13, *δ* = 0.39) (Figure 3e, Table 2). When breaking down the 5‐min segments of running, both sexes decreased equally over the first 10 min in the open field (time effect: *β* = −0.46, *q* < 0.001) (sex*time: *β* = 0.00, *q* = 0.99) (Figure 5f, Table 3). Over the final 20 min, males had a greater decrease in average running speed compared to females (time effect: *β* = −0.11, *q* < 0.001) (sex*time: *β* = 0.10, *q* = 0.004) (Figure 5f, Table 3).

##### Maximum (max) speed

Over the course of 30 min, males ran at a similar max speed (13.80 m/min ± 1.06) compared to females (14.29 m/min ± 1.21) (*q* = 0.087, *δ* = 0.43) (Figure 5g, Table 2). When examining 5‐min segments, both sexes decreased running equally over the first 10 min in the open field (time effect: *β* = −0.65, *q* < 0.008) (sex*time: *β* = 0.16, *q* = 0.73) (Figure 5h, Table 3). Over the final 20 min, males had a greater decrease in max running speed compared to females (time effect: *β* = −0.27, *q* < 0.001) (sex*time: *β* = 0.29, *q* = 0.001) (Figure 5f, Table 3).

##### Time in center

Over the course of 30 min in the open field, males spent a similar amount of time in the center of the testing box (14.5 m ± 4.3) compared to females (15.2% ± 4.8) (*q* = 0.74, *δ* = 0.14) (Table 2).

## 4 DISCUSSION

The current study shows that there are sex differences in outcome measures for these commonly used assays. Specifically, we show in voluntary wheel running, females ran greater distance and total time than males, consistent with prior studies. We also show that males and females initiate running bouts an equal number of times, but both average and max bout duration is greater in females, which likely contributes to greater distance and time run observed in females. In forced treadmill running, females ran for a significantly longer period but obtained the same top speed as males. In open field testing, there were no sex differences in total distance and speed, but males had significantly greater active time compared to females. Thus, each assay appears to be a unique measure of activity, with voluntary wheel running showing the largest sex‐specific differences. We previously showed that only about 40% of preclinical studies include both male and female rodents, and of those, only 17%–54% report sex differences (Plumb et al., 2023). Importantly, if these activity assays are being utilized to test new drugs or animal models, researchers should consider designing to test for sex differences as combining males and females could mask a unique effect in an individual sex.

### 4.1 Voluntary wheel running

Consideration of sex differences in wheel running behavior began in the early 1920's and numerous data support prominent sex differences in rodents; specifically, females run a greater distance and total time than males (Lightfoot, 2008; Lightfoot, 2013; Manzanares et al., 2018; Rosenfeld, 2017). The use of computer‐based data collection allows for a more comprehensive analysis of voluntary wheel running behavior (Greenwood & Fleshner, 2019). In agreement with prior literature performed in rats, we expanded these results by showing in mice a similar number of bouts between sexes, with females showing greater bout duration, greater speed, and greater rate of increase to plateau, all of which contribute to greater distance run (Basso & Morrell, 2017; Eikelboom & Mills, 1988; Tanner et al., 2023).

A bout is defined as a brief period of increased activity and has been considered an important component of physical activity (Bushman, 2020; WHO, 2024). For example, the American College of Sports Medicine recommends individuals accumulate 150 min of moderate‐to‐vigorous activity per week with bout durations of at least 10 min. However, clinicians often recommend increasing physical activity levels regardless of bout duration (Healey et al., 2012; Shirley et al., 2010) and prior research suggests improvements in function and pain, regardless of bout duration (Kehler et al., 2020; Yang, 2019). The current study showed a similar number of bouts per day between sexes but longer bout duration in females. Data were captured in 1‐min intervals, and bouts were separated by at least 1 min without running wheel activity. This agrees with previous research in rats where data was collected in 20‐s and 1‐min intervals (Eikelboom & Mills, 1988; Tanner et al., 2023). Conversely, in mice, De Bono et al. showed females ran a greater total number of bouts but showed no sex differences in bout duration—data were collected in 5‐s intervals, but it is unclear precisely how bouts were determined (De Bono et al., 2006). This difference is likely related to how bouts were collected and calculated and could represent the difference between a technical bout and a biologically meaningful bout of activity. Prior work in humans has shown that bouts of <10 min are associated with reduced frailty in both sexes (Kehler et al., 2020), but activity intensity is a greater determinant of cardiometabolic risk than bout duration (Tarp et al., 2018). Total activity time regardless of bouts is related to fatigue, function, and disease severity (Merriwether et al., 2018), while the total minutes spent in ≥10‐min bouts is associated with lower pain (Segura‐Jiménez et al., 2019) in individuals with fibromyalgia, suggesting both total activity and bouts are important in clinical populations. Future work in preclinical studies is needed to determine meaningful bout lengths.

The current study showed that both male and female mice reached a plateau after 2 weeks for daily distance but varied for other measures. Total running time normalized after 5 days, total bouts after 3 days, and average and peak bout duration between 8 and 14 days. These data are consistent with prior studies that showed that the speed of wheel running peaked within the 2 or 3 weeks in both sexes (Basso & Morrell, 2017; Konhilas et al., 2004), but contrast with others showing shorter durations for acclimation and more prominent sex differences (Bowen et al., 2016; De Bono et al., 2006). Differences could be related to the type of running wheel used, the external environment, train/species, or age of the animals.

Longer‐term acclimation is frequently performed to normalize wheel running. However, this is potentially problematic given the impact of voluntary wheel running, as a form of exercise, on physiological responses in multiple systems, including body composition, muscular system, metabolic capacity, peripheral and central nervous system, and immune system (D'Anci et al., 2000; Elias et al., 2023; Feldman‐Goriachnik et al., 2015; Kim et al., 2020; Lesnak, Berardi, & Sluka, 2023; Manzanares et al., 2018; Sluka et al., 2013; Sluka et al., 2020; Swallow et al., 1998). Physiological changes begin immediately with exercise and can produce biological effects within days (Cook et al., 2013; Feldman‐Goriachnik et al., 2015; Lesnak et al., 2022; Sluka et al., 2013). For example, running wheel activity in mice can prevent the development of chronic muscle pain and associated changes in the central nervous system with just 5 days of activity (Sluka et al., 2013). Therefore, if experimental mice undergo 2–3 weeks of wheel running to normalize running distance, it is likely these mice undergo physiological adaptations that alter the responses observed in otherwise sedentary mice. However, it could be argued that daily movement provides a better model of human behavior than limiting a rodent's movement by keeping them in a small cage. It likely depends on the level of active versus sedentary behavior in the human population being modeled.

### 4.2 Forced treadmill running

The current study showed females ran 1.5 min, 4.3%, longer than males. This difference was statistically significant, but whether the difference is biologically meaningful is debatable. As a secondary analysis, we included the animals that were available, but for perspective, at an effect size of 0.46 and an alpha of 0.05, we needed an *n* = 75 to achieve 0.8 power. The current study is in agreement with previously reported data, which shows females have greater time to exhaustion than males (Holcomb et al., 2022; Oydanich et al., 2019) while female rats run 40% less than males (Sun et al., 2018). Interestingly, these prior studies show larger effects, with female mice running 25%–50% longer than males. These differences are likely related to differences in the protocols, species (rat vs. mice), different treadmill inclines (10 vs., 20‐degrees), time spent at lower speeds, or rate of speed increase.

### 4.3 Open field testing

The open field, classically considered a test of emotionality and anxiety (Prut & Belzung, 2003; Ramos & Mormède, 1997), is also considered a good measure of exploration of a novel environment (Prut & Belzung, 2003). The current study showed no differences in activity between sexes for open field testing parameters of distance, speed, and percent of time in the center of the cage, but males had significantly longer active time. These data generally agree with prior studies in C57BL/6J mice that show equivalent activity between sexes (Fritz et al., 2017; Milner & Crabbe, 2008; Zeng et al., 2023). On the other hand, other mouse strains, MOLF and SJL, show higher activity in females (O'Leary et al., 2013). Interestingly, there were no differences in the percent of time in the center, suggesting no sex differences in anxiety in naïve mice. Complicating comparison between studies is differences in individual testing parameters, including size of the open field chamber, lighting, and the transparency of the walls (clear versus opaque). While the activity assessment of the 30‐min data in aggregate yielded no meaningful differences in distance and speed, a more in‐depth analysis examining data in 5‐min segments revealed unique sex‐specific differences. During the final 20 min of the test, females maintained running distance and speed similar to the first 10 min, while males showed steady decline during the last 20 min. This is a small but noteworthy effect, as it parallels the smaller bout duration in males that we see in voluntary wheel running relative to females.

### 4.4 Mechanisms of sex differences in activity assays

Sexually dimorphic differences in activity might be due to variation in sex hormones, muscle capacity, motivation, anxiety, or stress. Multiple studies show increases in estradiol enhance wheel running activity in male and female mice (Blizard, 1983; Gorzek et al., 2007; Greising et al., 2011; Lombard et al., 2019; Ogawa et al., 2003; Oydanich et al., 2019), but not exploratory behavior in the open field test (Levy et al., 2023; Miller & Meitzen, 2023; Zeng et al., 2023). Further, the sex differences in total distance, total duration, and speed of wheel running behavior disappear by 6–9 months of age (Bartling et al., 2017). Female mice show higher mobilization and use of lipids within the skeletal muscle than males and have more type I and less type II muscle fibers, which together could contribute to greater endurance (Holcomb et al., 2022; Oydanich et al., 2019). Thus, the sex‐specific differences likely involve multiple mechanisms across systems.

### 4.5 Strengths and limitations

This study was a secondary analysis of baseline data and thus was not designed specifically to test sex differences. However, protocols were consistent across animals in each cohort, and data was taken at baseline prior to any intervention. Separate mice were utilized for each assay; therefore, we were unable to correlate relationships within‐mouse between the different activity assays. Similarly, we did not account for muscle volume or cross‐sectional area. We collected wheel revolutions every minute. While common practice, extrapolating speed and bout data is less granular than previously reported by de Bono, who collected every 5 s (Prut & Belzung, 2003) and thus may be difficult to directly compare results between studies. Finally, the assays utilized, while being different measures of activity, also apply different stressors to the animals; therefore, it is possible that sex differences in activity are impacted by sex differences in the stress response to each individual assay. Animals receiving running wheels were individually housed on the first day of running. Social isolation is known to produce stress effects and could impact the running differently between sexes during the initial acclimation phase (Ieraci et al., 2016). The electric shocks utilized in treadmill running are a strong punishment stressor; however, we utilized a technique that uses prodding on testing day in place of electric shock shown to increase locomotion and reduce anxiety‐like behavior after testing (Khataei et al., 2021). The open field produces a short‐term social isolation stress as well as possible agoraphobia due to the size of the chamber (Prut & Belzung, 2003). Stress is an inherent part of all preclinical assays and warrants consideration.

### 4.6 Perspectives and significance

Taken together, important patterns in sex differences emerge from our 3 assays. Females show a slightly greater activity intensity as evidenced by differences in speed (smaller effect sizes), similar frequency of activity evidenced by total bouts, and a robust difference in the duration of activity evidenced by total distance (larger effect sizes). These differences were most pronounced for running wheel activity and did not translate to exploratory behavior in the open field test. Based on data from running wheel and treadmill tests, females appear to be less fatigable but have a similar maximum ability to males. The results in mice parallel research in humans showing females are less fatigable (Ansdell et al., 2020; Hunter, 2016; Lanning et al., 2017; Senefeld et al., 2013) and engage in more frequent moderate and light exercise than males (Craft et al., 2014). However, the parallels are not completely synchronous as human males demonstrate greater speed and power output (Jones et al., 2016). These data highlight the need to include both sexes in the research design, the need to disaggregate and analyze by sex, and the value of using multiple different assays to examine unique aspects of movement and activity.

## AUTHOR CONTRIBUTIONS

AJ conceived the idea, gathered, cleaned, and analyzed the data, and wrote the manuscript including tables and figures. GB, KH, and AP were involved in conceptualization, organization, presentation, and manuscript review. KH and JL recorded and gathered all running wheel data. TK recorded all forced treadmill data and reviewed the manuscript. BM contributed to open field data recording and analysis. All experiments were performed in the labs of KS and CB, who both contributed significantly to the conceptualization of experimental design and analysis and preparation and manuscript review. Identify which authors participated in the research: Conceived and designed research, performed experiments, analyzed data, interpreted results of experiments, prepared figures, drafted manuscript, edited and revised manuscript, approved final version of manuscript. The information must be the same as in the online submission site.

## FUNDING INFORMATION

This study was supported by National Institutes of Health, R01AR073187 to KAS; U.S. Department of Veterans Affairs, 5I01BX000776 to CJB; Foundation for Physical Therapy Research PODS I & II to AJJ.

## CONFLICT OF INTEREST STATEMENT

The authors have no perceived or potential conflicts of interest.

## ETHICS STATEMENT

All experiments were approved by the University of Iowa Animal Care and Use Committee and were conducted in accordance with the National Institute of Health's Guidelines for the Care and Use of Laboratory Animals.

## Data Availability

The data sets supporting the conclusion are available from the corresponding author upon request.
